# Evolutionarily conserved neural responses to affective touch in monkeys transcend consciousness and change with age

**DOI:** 10.1073/pnas.2322157121

**Published:** 2024-04-22

**Authors:** Joey A. Charbonneau, Anthony C. Santistevan, Erika P. Raven, Jeffrey L. Bennett, Brian E. Russ, Eliza Bliss-Moreau

**Affiliations:** ^a^Neuroscience Graduate Program, University of California, Davis, CA 95616; ^b^Neuroscience and Behavior Unit, California National Primate Research Center, University of California, Davis, CA 95616; ^c^Department of Psychology, University of California, Davis, CA 95616; ^d^Department of Radiology, Center for Biomedical Imaging, New York University Grossman School of Medicine, New York, NY 10016; ^e^Department of Psychiatry and Behavioral Sciences, University of California, Davis School of Medicine, Sacramento, CA 95817; ^f^The Medical Investigation of Neurodevelopmental Disorders Institute, University of California, Sacramento, CA 95817; ^g^Center for Biomedical Imaging and Neuromodulation, Nathan Kline Institute, Orangeburg, NY 10962; ^h^Nash Family Department of Neuroscience, Icahn School of Medicine at Mount Sinai, New York, NY 10029; ^i^Friedman Brain Institute, Icahn School of Medicine at Mount Sinai, New York, NY 10029; ^J^Department of Psychiatry, New York University Langone, New York, NY 10016

**Keywords:** affective touch, rhesus monkey, insula, interoception, aging

## Abstract

Affective touch—a slow, gentle, and pleasant form of touch—activates a different neural network than which is activated during discriminative touch in humans. Affective touch perception is enabled by specialized low-threshold mechanoreceptors in the skin with unmyelinated fibers called C tactile (CT) afferents. These CT afferents are conserved across mammalian species, including macaque monkeys. However, it is unknown whether the neural representation of affective touch is the same across species and whether affective touch’s capacity to activate the hubs of the brain that compute socioaffective information requires conscious perception. Here, we used functional MRI to assess the preferential activation of neural hubs by slow (affective) vs. fast (discriminative) touch in anesthetized rhesus monkeys (*Macaca mulatta*). The insula, anterior cingulate cortex (ACC), amygdala, and secondary somatosensory cortex were all significantly more active during slow touch relative to fast touch, suggesting homologous activation of the interoceptive-allostatic network across primate species during affective touch. Further, we found that neural responses to affective vs. discriminative touch in the insula and ACC (the primary cortical hubs for interoceptive processing) changed significantly with age. Insula and ACC in younger animals differentiated between slow and fast touch, while activity was comparable between conditions for aged monkeys (equivalent to >70 y in humans). These results, together with prior studies establishing conserved peripheral nervous system mechanisms of affective touch transduction, suggest that neural responses to affective touch are evolutionarily conserved in monkeys, significantly impacted in old age, and do not necessitate conscious experience of touch.

## Significance

Affective touch is thought to be a critical substrate for the formation of the social relationships which exist as a foundation for primate societies. Although grooming behavior in monkeys appears to recapitulate features of affective touch behavior in humans, it is unknown whether affective touch activates the same neural networks in other primate species and whether this activation requires conscious perception or changes across the lifespan. We stimulated lightly anesthetized macaques at affective (slow) and discriminative (fast) touch speeds during the acquisition of functional MRI data. We demonstrate evolutionarily conserved activation of interoceptive neural networks which change significantly in old age.

Whether the comforting caress of an infant by a parent or the reassuring rub between close friends, interpersonal tactile experiences are a core feature of our socioaffective lives. This slow, gentle touch—termed “affective touch” (1)—plays a critical role in normative human development (2) and may be fundamental to the unique social relationships—and social brains—of primates (3). Affective touch sensation is enabled by a specialized class of low threshold mechanoreceptors with unmyelinated fibers called C tactile (CT) afferents (4, 5) which selectively innervate the hairy skin of humans (1) and other mammals [e.g., cats (6); monkeys (5)]. They are preferentially activated by touch speeds between 1 and 10 cm/s (7)—a speed which elicits “pleasant” sensations in people (8). Given the slow conduction velocity of the unmyelinated fibers conveying affective touch (0.5 to 2 m/s), these afferents are thought to serve a categorically different role in the processing of touch-related sensory information as compared to the myelinated fibers which quickly (20 to 80 m/s) convey discriminative touch information used to detect and identify external stimuli influencing rapid decision-making processes and guiding behavior (7). CT afferents also project to different cortical targets from those conveying discriminative touch, activating a dissociable (9) network of neural hubs related to the generation of affective states, grounded in the insula (10). For these reasons, affective touch has come to be widely considered an interoceptive—rather than exteroceptive—sensory modality (7, 11–13).

Several human neuroimaging studies provide insight into the neural networks responsible for processing affective touch (10, 14–16). Although selective activation of unmyelinated CT fibers is typically impossible without parallel activation of myelinated afferents, evidence from a unique patient lacking large diameter myelinated afferents showed that CT fiber stimulation results in activation of the posterior insula, but not somatosensory cortex (10). Further, a meta-analysis of neural responses to affective and discriminative touch found that while affective touch preferentially activated right posterior insula, discriminative touch activated primary somatosensory cortex, and both activated secondary somatosensory cortex in the parietal operculum (9). Affective touch also activates other neural hubs thought to be particularly important for the processing of affective and social information, including dorsal anterior cingulate cortex (ACC), orbitofrontal cortex, superior temporal sulcus, and amygdala (14, 17–19). These hubs belong to the interoceptive-allostatic network (IAN), a replicable large-scale intrinsic network in the human brain for interoceptive processing and allostatic prediction (20).

There has been much theorizing about the critical role that affective touch plays in primate societies by supporting the generation and maintenance of social relationships (e.g., refs. 3 and 21–23) and there is evidence that affective touch plays important roles in several dimensions of human social behavior (24–27). Affective touch is one means by which nonhuman primates form and nurture social bonds (23). Additionally, the speed at which monkeys move their hands while grooming falls within the range of optimal speeds for triggering CT afferents (28). However, there is currently very little evidence supporting the evolutionary conservation of neural mechanisms underlying affective touch. Neurophysiological recordings from the posterior insula of a single macaque monkey suggest that some neurons in this region may respond to affective touch (29) and, just as observed in human infants (30, 31) and adults (32, 33), affective touch of two macaque monkeys by a familiar human experimenter resulted in a decrease in heart rate and increase in heart rate variability (34). Further, the amygdala—which is heavily interconnected with the insula—shifted its baseline firing when human experimenters groomed macaque faces suggesting that its neurons may propagate information about social context through the IAN (35). These data suggest that a similar network may be encoding affective touch in humans and monkeys, but whether whole brain networks responsive to CT fiber-mediated touch in humans are present in other species is unknown. It also remains unclear whether the networks activated by affective touch in humans are a product of the strict sensory inputs or require the conscious representation of that sensory information.

In the present study, we evaluated the evolutionary origins of neural responses to affective touch by measuring brain activity during affective vs. discriminative touch in adult rhesus monkeys using functional MRI (fMRI). Monkeys were lightly anesthetized with isoflurane during the procedure, which also allowed us to evaluate whether neural responses to affective touch require conscious representation. By utilizing anesthesia, we were able to collect data from a very large sample (N = 33) of rhesus monkeys for a task-based fMRI experiment.

## Results

### Insula Is Preferentially Responsive to Affective Touch.

**Fig. 1.**
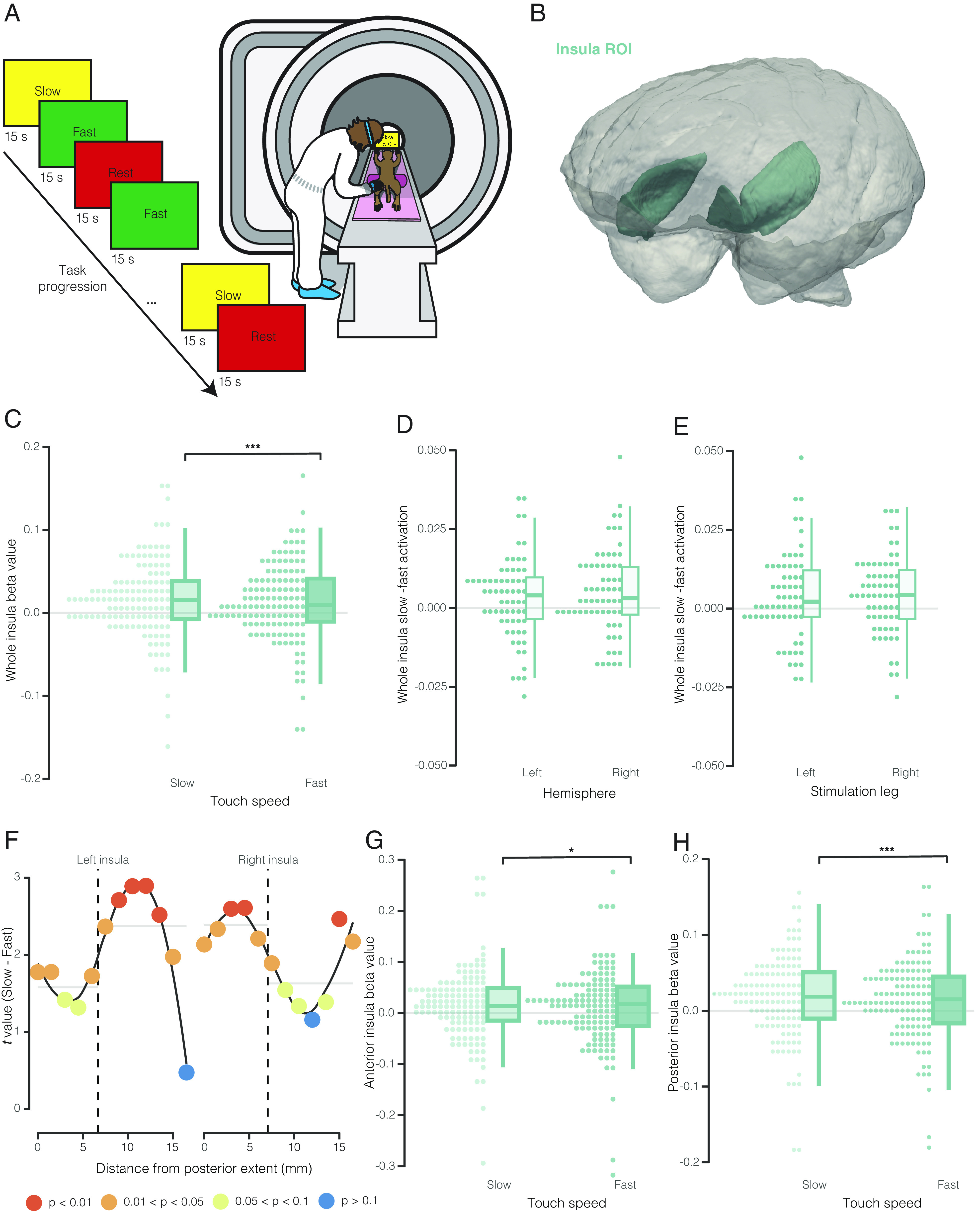
Insula activation by affective vs. discriminative touch. (*A*) Schematic representation of the affective touch paradigm, showing trial sequence (*Left*) and testing set up in the MRI scanner (*Right*). (*B*) 3D rendering of the insula region of interest used for analyses embedded in a 3D representation of the NIMH macaque template reference brain. (*C*) Comparison of whole insula activation (beta values) during slow (affective) vs. fast (discriminative) touch conditions. Individual data points represent beta values for left and right insula for each subject (2 points/subject in each condition). Boxes in the box plots show the 25th, 50th, and 75th percentiles. Whiskers show the maximum and minimum points within 1.5× the interquartile range. At the group level, slow touch activation was significantly greater than fast touch activation. (*D* and *E*) Comparison of the difference in beta values for each subject across insula hemispheres (*D*) and leg of stimulation (*E*). Each data point shows the difference score for an individual subject (1 point/subject for each hemisphere/leg). At the group level, difference scores differed significantly from 0 in both hemispheres and both legs. (*F*) *t*-statistics comparing group-level activation during slow vs. fast conditions in sliding regions of interest throughout the anterior–posterior extent of the insula. *P*-values are colored according to level of significance. Left insula data are shown on the left, and right insula data are shown on the *Right*. Vertical dashed lines show the change points detected with change point analysis. Horizontal gray lines show the mean *t*-statistic for each segment after division according to change points detected. (*G* and *H*) Comparison of activation in the anterior (*G*) and posterior (*H*) insula during slow vs. fast touch conditions. As in *C*, individual data points represent beta values for left and right insula for each subject (2 points/subject in each condition). In both insula subregions slow touch activation was significantly higher than fast touch activation at the group level.

Thirty-three adult rhesus macaques (*Macaca mulatta*) of both sexes (N = 23 female) were subjected to an affective touch paradigm while undergoing fMRI scanning. During 15-s blocks, they received slow (3 cm/s; the affective condition), fast (15 cm/s; the discriminative condition), or no touch stimulation to their outer thigh by a gloved experimenter (Fig. 1*A*). Insula—specifically, the posterior granular portion—is the canonical site of primary interoceptive-sensory processing in the primate brain (11, 36, 37). Insula, along with a network of socioaffective neural hubs, is preferentially activated by slow vs. fast (affective vs. discriminative) touch in humans (9). As such, our primary contrast was the difference between the slow and fast conditions, revealing neural responses to interoceptive affective touch above and beyond neural responses to exteroceptive discriminative touch.

We found a significant effect of touch speed on neural activation in the monkey insula (χ^2^(1) = 12.22, *P* = 0.0005) (Fig. 1*B*). Insula was significantly more active during slow touch [estimated marginal mean (EMM) = 0.016] than during fast touch (EMM = 0.012) (Fig. 1*C*). Generalized linear mixed models including insula hemisphere (left vs. right) and leg of stimulation (left vs. right) showed no effect of either variable on insula activation nor interactions with touch speed, indicating that preferential activation of the insula in response to slow touch was bilateral and indifferent to stimulation side (Fig. 1 *D* and *E*).

Insula exhibits considerable heterogeneity in both structure and function across humans and nonhuman primates (37–39). In particular, the posterior portion of the insula appears to be specifically and preferentially activated by slow touch in humans (9). Given the large size of our insula ROI (regions of interest), it remained possible that preferential responsivity to slow touch was restricted to just one portion. In humans, insula can be divided into anterior and posterior subregions on the basis of the central insular sulcus but that landmark is not present in monkeys. Rather than assign an arbitrary boundary to divide insula on the anterior–posterior (A–P) axis, we assessed the gradient of activation to slow vs. fast touch using a sliding region of interest (four voxels wide on the A–P axis, sliding by one voxel) throughout the extent of the insula (*SI Appendix*, Fig. S1*A*). This approach allowed us to assess the profile of the difference in insula activation by slow vs. fast touch along the A–P axis. Changepoint analyses revealed an inflection point ~7 mm from the posterior extent of insula in both hemispheres. Using this boundary, we then evaluated slow vs. fast touch in the anterior and posterior subregions separately (*SI Appendix*, Fig. S2 *A* and *B*). Both functionally defined anterior and posterior subregions of insula were significantly more active during slow touch (anterior: χ^2^(1) = 5.97, *P* = 0.01, posterior: χ^2^(1) = 11.34, *P* = 0.0008) (Fig. 1 *G* and *H*). Interestingly, this analysis revealed different patterns of activation across the left and right hemispheres—there was a larger difference in slow vs. fast touch activation in the anterior portion of the left insula and the posterior portion of the right insula (Fig. 1*F* and *SI Appendix*, Fig. S1 *B* and *C*). However, the effect of hemisphere was not statistically significant when we separately evaluated slow vs. fast touch activation in the anterior and posterior subregions, likely due to the fact that while there was a more dramatic difference in slow vs. fast touch activation in the right posterior and left anterior subregions, the pattern of activation was still the same throughout the entire structure (*SI Appendix*, Fig. S2 *C* and *D*). We did not find any evidence of differences between males’ and females’ whole insula, anterior subregion, and posterior subregion responses to slow vs. fast touch (*SI Appendix*, Fig. S3 *A*–*C*).

### Network Representation of Slow Touch in ACC and Amygdala.

**Fig. 2.**
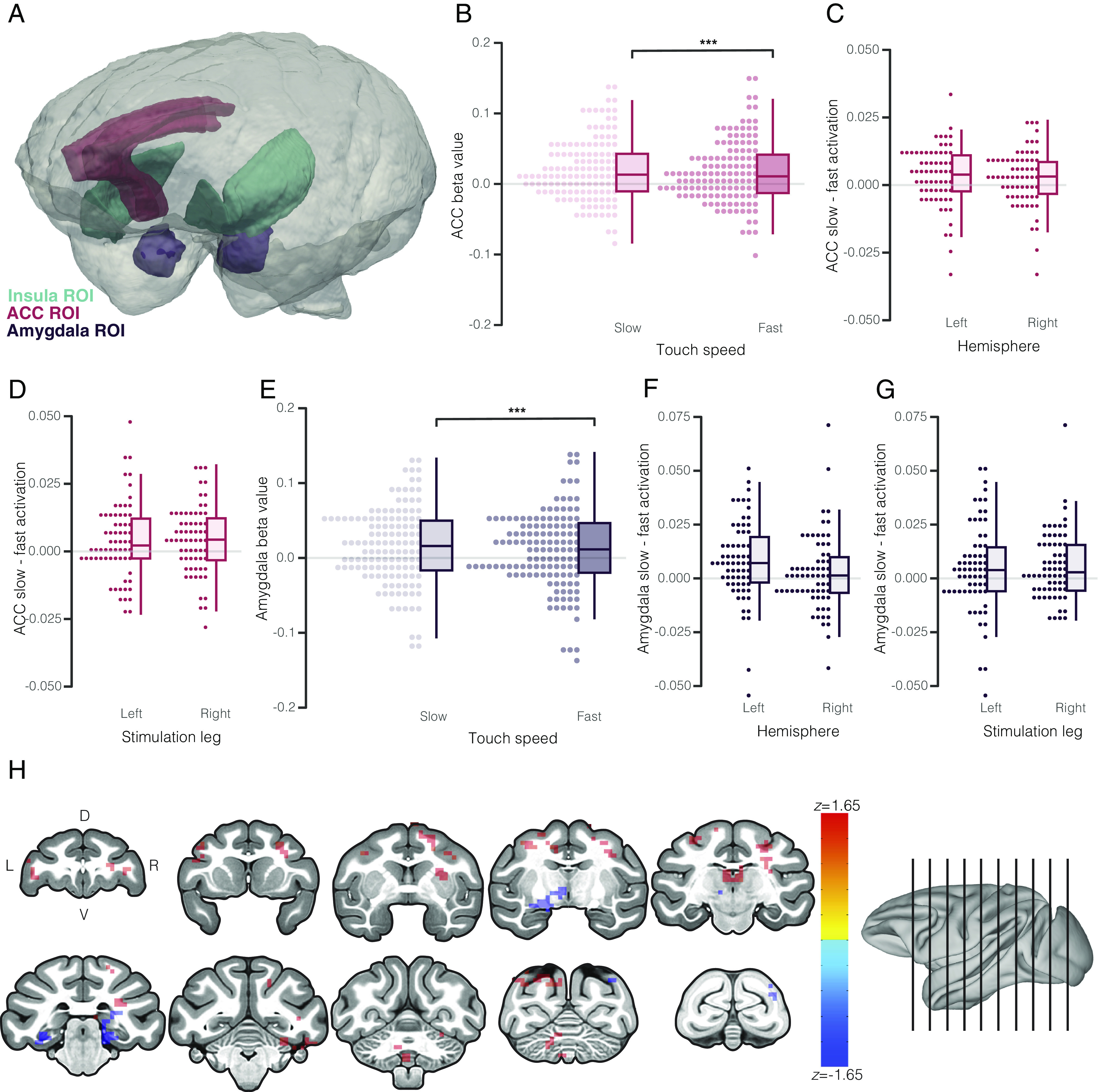
Activation of other IAN hubs by affective vs. discriminative touch. (*A*) 3D renderings of insula (teal), ACC (red), and amygdala (purple) ROI used for analyses embedded in a 3D representation of the NIMH macaque template reference brain. (*B*) Comparison of ACC activation (beta values) during slow (affective) vs. fast (discriminative) touch conditions. Individual data points represent beta values for left and right ACC for each subject (2 points/subject in each condition). Boxes in the box plots show the 25th, 50th, and 75th percentiles. Whiskers show the maximum and minimum points within 1.5× the interquartile range. At the group level, slow touch activation was significantly greater than fast touch activation. (*C* and *D*) Comparison of the difference in beta values (slow–fast) for each subject across ACC hemispheres (*C*) and leg of stimulation (*D*). Each data point shows the difference score for an individual subject (1 point/subject for each hemisphere/leg). At the group level, difference scores differed significantly from 0 in both hemispheres and both legs. (*E*) Comparison of amygdala activation during slow (affective) vs. fast (discriminative) touch conditions. (*F* and *G*) Comparison of the difference in beta values (slow–fast) for each subject across amygdala hemispheres (*F*) and leg of stimulation (*G*). The difference between slow and fast activation was significantly different from 0 in the left, but not right, amygdala. Amygdala activation by slow vs. fast touch was significantly different from 0 for both left and right leg stimulation. (*H*) Representative coronal sections demonstrating task-related shifts in posterior insula network connectivity revealed by psychophysiological interaction analysis.

Despite the fact that there is very little interoception research in nonhuman primates (although see refs. 40–42), neural models of interoceptive processing in humans (20, 43) have been constructed on the basis of macaque anatomical studies (44–52). When one such model [the Embodied Predictive Interoceptive Coding model (43)] was experimentally tested, it revealed the presence of the IAN. The IAN is composed of two main subnetworks, the salience (SN) and default mode networks (DMN)—networks which exist, as measured by resting state fMRI, in both humans [SN (53); DMN (54)] and monkeys [SN (55); DMN (56)]. In addition to insula, the other core regions in the IAN are ACC and amygdala (Fig. 2*A*)—which have been implicated in the encoding of social and interoceptive signals by intracranial recording studies in macaques (35, 40).

In the current study, ACC was also preferentially activated by slow vs. fast touch (χ^2^(1) = 10.20, *P* = 0.001) (Fig. 2*B*). As in previous analyses, there was no effect of hemisphere or stimulation side, indicating a bilateral response regardless of which leg was touched (Fig. 2 *C* and *D*). Mean betas during the slow (EMM = 0.020) and fast (EMM = 0.017) conditions were generally higher in ACC than in insula across both conditions, but the difference between conditions was similar to difference seen in insula.

Slow touch also activated the amygdala significantly more than fast touch (χ^2^(1) = 10.93, *P* = 0.0009) (Fig. 2*E*). Analysis of amygdala activation suggested potentially lateralized responding (touch speed × hemisphere: χ^2^(1) = 3.35, *P* = 0.067). Post hoc analyses revealed a significant difference between slow and fast activation in the left (*P* = 0.005) but not the right (*P* = 0.75) amygdala (Fig. 2 *F* and *G*). In the left amygdala, activation by slow touch was elevated (EMM = 0.022) relative to fast touch (EMM = 0.014) and in the right amygdala activation was low in both conditions (slow: EMM = 0.015, fast: EMM = 0.012). Thus, while cortical processing of interoceptive touch was bilateral, subcortical processing of these signals in the amygdala was left hemisphere-specific. As in insula, we did not find any evidence of sex differences in activation of the ACC or amygdala by slow vs. fast touch (*SI Appendix*, Fig. S3 *D* and *E*).

To further characterize system interactions elicited by slow touch, we carried out a psychophysiological interaction (PPI) analysis (57, 58), which has the ability to reveal task-based differences in network connectivity (above and beyond independent changes in neural activity in different regions) using a seed-based approach. When the posterior insula was seeded, slow touch increased connectivity with targets in sensorimotor regions (primary and secondary somatosensory, primary motor, and premotor cortex, putamen, and cerebellum) as well as superior temporal regions (including superior temporal sulcus and gyrus) and thalamus. Slow touch also decreased connectivity with the hippocampus and parahippocampal cortex (Fig. 2*H*). Notably, changes in connectivity with anterior insula, ACC, and amygdala were not present in the results of this analysis, potentially indicating independent interoceptive processing across these hubs or disruption of what would be coordinated activity in this network by the anesthetic agent.

### Activation beyond the IAN.

We anticipated that if the neural representation of affective touch was similar between humans and monkeys, we would not only see preferential activation by slow touch of interoceptive hubs like insula, ACC, and amygdala but also specific activation of somatosensory regions—namely, secondary somatosensory cortex (SII). Studies in humans suggest that SII plays an important and potentially specific role in processing affective touch (18, 59) and other interoceptive signals (60), while primary somatosensory cortex (SI) is more involved in the processing of discriminative touch (9).

**Fig. 3.**
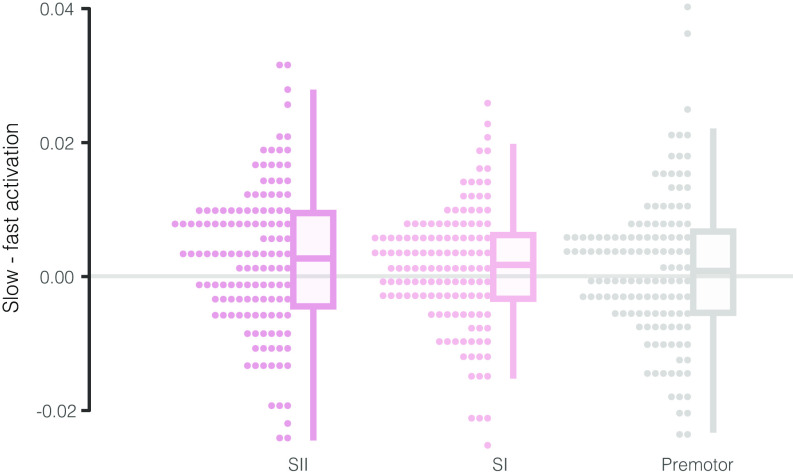
The difference in beta values during slow vs. fast touch conditions for secondary somatosensory cortex (*Left*, dark pink), primary somatosensory cortex (*Middle*, light pink), and premotor cortex (*Right*, gray). The difference between slow and fast touch activation within subject differed significantly from 0 only in secondary somatosensory cortex. Positive values indicate greater activation by slow touch and negative values indicate greater activation by fast touch. Boxes in the box plots show the 25th, 50th, and 75th percentiles. Whiskers show the maximum and minimum points within 1.5× the interquartile range.

As predicted, there was a significant effect of touch speed in SII (χ^2^(1) = 7.94, *P* = 0.005). SII was significantly more active during slow (EMM = 0.017) as compared to fast (EMM = 0.015) touch (Fig. 3). There was no interaction between touch speed and hemisphere or stimulation leg in SII. Additionally, there was no effect of touch speed on activity in SI (χ^2^(1) = 3.13, *P* = 0.08). There was a significant effect of stimulation leg (χ^2^(1) = 5.30, *P* = 0.02), such that activation was significantly greater regardless of touch speed when stimulation was applied to the right vs. left leg (right: EMM = 0.028, left: EMM = 0.014). These results are consistent with the idea that while SI is more generally involved in processing tactile sensory information (and therefore would represent slow and fast touch similarly), SII may be better tuned to specifically handle affective touch and potentially other interoceptive stimuli (Fig. 3). Whole brain activation data are shown in *SI Appendix*, Fig. S4.

We assessed activation of premotor cortex adjacent to SII as a control region that we did not expect to differentially respond to slow vs. fast touch. There were no differences in activation between the slow and fast touch conditions (χ^2^(1) =1 .35, *P* = 0.25) (Fig. 3). This suggests that the effect of touch speed on neural activation is specific to interoceptive and somatosensory regions, consistent with the evidence available from human imaging experiments.

### Neural Responses to Affective Touch Change in Old Age.

Our ROI-based analyses made it clear that there were considerable individual differences in the extent to which the IAN was preferentially activated by slow (affective) touch (Figs. 1*C* and 2 *B* and *E*) which were not attributable to sex differences. The monkeys in our sample spanned a wide age range from early middle adulthood (aged 7 y; equivalent to mid-to-late 20 s in humans) through old age (aged 20 y; equivalent to 70 to 80 in humans). Given the substantial literature on the loss of myelinated and unmyelinated nerve fibers in aged people (see ref. 61 for a review), the substantial literature documenting age-related changes in human interoceptive processing (e.g., refs. 62–64), and the likely implications of this for central nervous system processing of affective touch, we assessed the impact of age on touch responding in our sample.

**Fig. 4.**
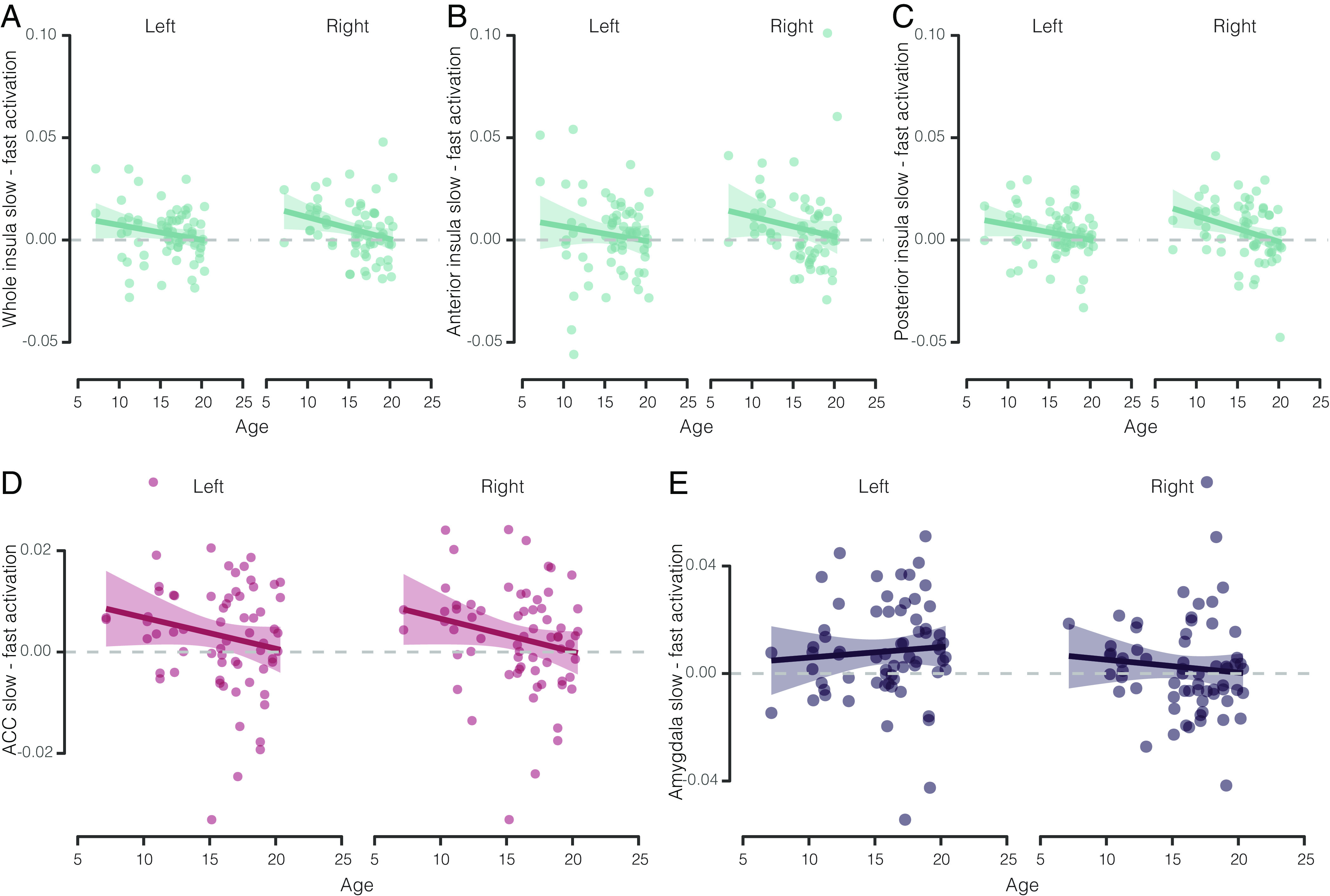
(*A*–*E*) Difference in beta values during slow vs. fast touch conditions as a function of age for whole insula (*A*), anterior insula (*B*), posterior insula (*C*), ACC (*D*), and amygdala (*E*). For all cortical ROI (*A*–*D*), there was a significant decrease in preferential activation by slow touch with age. For amygdala (*E*), there was no significant age-related change. In all cases, left vs. right hemisphere structures are shown separately. Individual data points show the difference in activation across the ROI for slow–fast conditions.

There was a significant interaction between touch speed and age (modeled as a continuous variable) on activation in the whole insula ROI (χ^2^(1) = 7.21, *P* = 0.007) (Fig. 4*A*). To evaluate this effect, we carried out post hoc tests using EMMs computed for animals at 10, 15, and 20 y of age. While at ages 10 and 15, there was a significantly greater response to slow vs. fast touch (both *P* = 0.001), by age 20 there was no longer a significant difference between conditions (*P* = 1.00). Notably, at age 10, activation to slow touch in insula was high (EMM = 0.018) while activation to fast touch was low (EMM = 0.008), and at age 20, activation during both conditions was high (slow: EMM = 0.015, fast: EMM = 0.015). This pattern of effects suggests that the mechanism underlying changes in human experiences of affective touch with age [i.e., decreased intensity but increased pleasantness (65)] may not be the result of reduced sensitivity to slow touch but instead equilibration of insula’s representation of slow and fast touch. When the anterior and posterior portions of insula were considered separately (using our functionally derived ROIs), there was the same touch speed × age interaction in the posterior insula (χ^2^(9.33) = 9.33, *P* = 0.002) (Fig. 4*B*), but not in the anterior insula (χ^2^(1) = 2.82, *P* = 0.09) (Fig. 4*C*). This suggests that changes in posterior insula may underlie the age-related shift seen at the level of the whole insula, rendering this specific region an important target of interoceptive aging research.

In the ACC, we also found a significant interaction between touch speed and age (χ^2^(1) = 5.30, *P* = 0.02) (Fig. 4*D*). Unlike in insula, the interaction in ACC was not driven by age-related increases in activation to fast touch. Rather, while younger animals (age 10) had higher activation during slow touch (EMM = 0.028) relative to lower activation during fast touch (EMM = 0.021), older animals (age 20) showed reduced activation to both stimuli (slow: EMM = 0.014, fast: EMM = 0.014). This may be indicative of different mechanisms of age-related psychophysiological changes in the ACC and insula.

In the amygdala, there was no interaction between touch speed and age (χ^2^(1) = 0.0087, *P* = 0.93) (Fig. 4*E*). This was true even when only left amygdala, where we did detect a significant effect of touch speed, was considered (χ^2^(1) = 0.32, *P* = 0.57), suggesting that age-related changes in interoceptive responding to touch may be restricted to the cortical hubs processing this stimulation. We also did not find any significant interaction between touch speed and age in SII (χ^2^(1) = 1.55, *P* = 0.21) or SI (χ^2^(1) = 0.56, *P* = 0.45), suggesting that age-related changes in affective touch processing may be specific to cortical primary interoceptive hubs, sparing somatosensory hubs (*SI Appendix*, Fig. S5).

## Discussion

We demonstrate that monkeys and humans share a neural mechanism that responds to slow, C-fiber stimulating touch. In monkeys, just as in people (9, 14), this slow, “affective”^*^ touch elicited significantly greater activation of interoceptive and socioaffective neural hubs than did discriminative touch—even under anesthesia. Affective touch selectively activated hubs including insula, ACC, amygdala, and secondary somatosensory cortex—all of which have been implicated in affective touch representation in humans (14, 17, 18, 21). This pattern of activation in both monkeys and humans suggests comparable and evolutionarily conserved central nervous system mechanisms of affective touch. These data build on a decades-old literature demonstrating the presence of similar peripheral nervous system physiology, including the presence of unmyelinated CT afferents in the skin of macaques (5) and humans (66) which project to the superficial layers of the spinal dorsal horn (67, 68) as well as recent single-cell RNA sequencing evidence indicating evolutionary conservation of CT fibers (or C-low threshold mechanoreceptors) with similar transcriptomic profiles across humans and macaques (69).

In addition to preferential activation of IAN hubs by slow touch, we found that primary somatosensory cortex (SI) was activated by both affective and discriminative touch. Although meta-analysis of human task fMRI studies suggests that primary somatosensory cortex is specifically and preferentially activated by discriminative touch (9), there is evidence to suggest that SI may also play an important role in responding to the affective components of touch. For example, when heterosexual male participants experienced affective touch from a single experimenter, whether they were shown a male or female individual engaging in the caress during this touch significantly modulated responses in SI (70). Given that our subjects were under anesthesia, it seems unlikely that social context—or perceived social context—modulated neural responses in our sample. It remains possible, however, that in the absence of such social cues, there is a lack of inhibition or modulation of SI responses which might lead to differential activation across conditions. Further, it may be the case that our selected touch speeds, 3 cm/s and 15 cm/s, were not sufficiently different to drive differential responding in SI. Faster touch speeds (e.g., 30 cm/s in ref. 71) have been used for discriminative touch conditions in human studies, which may provide a better contrast. Likewise, early work in monkeys indicated that very slow touch velocities may optimally activate CT fibers (5), which might also increase the contrast in neural activation between conditions. Future work should characterize the psychometrics of different touch speeds to determine optimal speeds across conditions and provide a basis for comparison of macaques to humans.

Establishing a nonhuman primate model of affective touch provides a foundation for causal investigations of the peripheral and central mechanisms of affective touch transduction, representation, and perception, as well as how this touch generates and maintains social bonds critical for primate societies (3, 7, 23). That is, in future study, we will have the opportunity to manipulate monkeys’ physiology (e.g., inactivation or ablation of peripheral nerves or neural hubs) and, or, their social context (e.g., social network size, the demographic nature of available social partners) to determine the precise links between monkeys’—and, given the homology, humans’—internal and external worlds. Nonhuman primates may also be critically important to future translational research on disorders in which affective touch perception is perturbed, including autism spectrum disorders (72–74) [for which nonhuman primate models are likely to offer superior translational potential relative to other preclinical models like rodents (75, 76)] and eating disorders (77–79).

Our results also demonstrate robust age-related variation in the brain’s responsiveness to affective touch. Rhesus monkeys age 3 to 4 times faster than humans, reaching reproductive age around 3 to 5 y old and old age around 18 y of age (80). Our sample, ages 7 to 20 (corresponding to ~25 to 80 human years) covered a large portion of adulthood. Younger monkeys showed greater activation of posterior insula and ACC by affective vs. discriminative touch and in older monkeys these regions were active to the same degree during both conditions. We observed no age-related differences in activation in somatosensory cortex or amygdala, suggesting specific age-related changes in ACC and insula.

Studies of affective touch in older people are rare. In a recent meta-analysis, only 3 studies (3% of those reviewed) evaluated affective touch in people over 60 y of age (and only narrowly above this threshold, between 60 and 64 y on average) (81). One of these studies found that with age, the perceived intensity of all touch decreased significantly, while the perceived pleasantness increased (65). One explanation is that intensity is mediated by peripheral afferent density—known to decline with age (61)—while pleasantness is mediated by top–down mechanisms. Cortical interoceptive hubs may adapt with age promoting positive valence responses to affective (and, potentially, nonaffective) stimuli (7, 65). This may explain why our age-related changes were specific to socioaffective regions and excluded somatosensory regions. Age-related changes in posterior insula were driven by increased activation to both affective and discriminative touch, such that there was no longer a difference between conditions. This may suggest a potential mechanism wherein representation of incoming tactile stimulation is amplified in the posterior insula of aging brains—likely via some combination of recurrent thalamocortical and corticocortical circuity. Additionally, the reduced ACC activation by both stimulation types could be explained by a decreased number of projections between these regions as synapses degrade in aging brains (see ref. 82 for a review of prefrontal cortical aging in macaques). Future investigations of this circuitry in monkeys, including mechanistic pathway-specific manipulations (e.g., inactivation of IAN hubs during touch stimulation), can elucidate the underlying causes of age-related changes. Further, we can accelerate cortical aging in monkeys, like the induction of early-stage Alzheimer’s disease (AD)-like pathology (83), and assess similarities to healthy aging subjects, potentially revealing important early disease markers measurable with noninvasive tools. Work on affective touch in this realm is likely to be particularly informative given that there are established functional and structural changes to insula in AD (84), including cytoarchitecturally dependent accumulation of neuritic plaques and neurofibrillary tangles (85), selective gray matter volume loss (86), and subregion-specific shifts in resting state functional connectivity which may precede obvious symptomology (87).

The present work demonstrates that IAN neural responses to affective touch transcend species and consciousness—given that monkeys were anesthetized. Questions remain, however, about how slow, C-fiber stimulating touch becomes affective. People report slow touch as more pleasant than discriminative touch, providing a basis for the label affective touch (1). In people, processing of conscious feeling states is thought to be accomplished, in part, by the anterior insula, which may gate network transitions allowing for primary sensory information, like that received by posterior insula, to reach consciousness (88). Our PPI analysis did not find task-evoked changes in functional connectivity between posterior insula and anterior insula during the slow vs. fast touch conditions. This is in contrast with findings in humans, which indicate that stroking of the skin increased functional connectivity between the posterior and ventral anterior insula (89). This difference could be attributable to disruption of intrainsular network representation of touch (i.e., the forward-downward cascade with recurrent connectivity) by anesthesia or species differences resulting from the expansion of the anterior insula in humans (90).

We did, however, find evidence of preferential activation in the anterior insula during slow vs. fast touch suggesting that the neural instantiation of affective feelings during slow touch may not require consciousness and that anterior insula activity is not sufficient to produce conscious experience. Anterior insula is consistently implicated in processes related to consciousness (88, 91, 92) and particularly awareness of the self (93), in part because of the presence of Von Economo neurons which have been proposed as a substrate of consciousness (94, 95) and are present in humans and macaques (94, 96). Future research investigating the extent to which different levels of consciousness—induced, for example, by varied levels of anesthesia or different anesthetic agents—impact insula activation and intrainsular circuitry during affective touch and other tasks may provide insights about the nature of the biology (and philosophy) of consciousness. Further, although replicating the present study in awake monkeys will not be feasible in a comparable sample size, further research analyzing task-evoked changes to functional connectivity in awake monkeys would be of great value.

Monkeys cannot verbally report on their conscious experiences nor provide verbal ratings of the pleasantness of various types of tactile stimulation. However, it is possible to index the “pleasantness” of their experience via indirect means in future studies. We have consistently demonstrated that respiratory sinus arrhythmia (RSA; high-frequency heart rate variability) tracks with affective valence in monkeys such that when pleasant states are induced, RSA is higher than during neutral or negative states (97, 98). An increase in RSA during slow vs. fast touch in monkeys, as observed in humans (33), would provide further evidence that monkeys experience slow touch as being pleasant, like people do. We did carry out physiological monitoring during the scans for the purposes of anesthesia monitoring but the data were not sufficient to determine variation in RSA on a trial-by-trial basis—a limitation of the present report and clear direction for future research. However, if preferential activation of insula by slow vs. fast touch is affective and related to experiences of pleasantness, then our data also demonstrate that affect need not be conscious, consistent with decades of theorizing on the nature of affect in people (99–102). Further, understanding age-related differences in the pleasantness of affective touch will open opportunities to study affective aging trajectories, develop treatments and interventions for age-related diseases impacting affective processing, and understand how conscious experiences of pleasantness vary across the lifespan.

## Materials and Methods

All experimental protocols were approved by the University of California Davis Institutional Animal Care and Use Committee and carried out in accordance with the US NIH guidelines. All procedures were carried out at the California National Primate Research Center (CNPRC).

### Subjects.

Subjects were 33 adult rhesus macaques (*M. mulatta*) aged 7.15 to 20.35 y (mean ± SD = 15.81 ± 3.42) and weighing 5.57 to 20.40 kg (mean ± SD = 11.14 ± 3.88). Ten subjects were male (age: 17.57 ± 1.24 y, weight: 15.05 ± 3.79 kg), and 23 subjects were female (age: 16.21 ± 2.37 y, weight: 8.93 ± 1.68 kg). All monkeys were born and raised at the CNPRC. Monkeys were raised outdoors in small (housing 10 to 30 monkeys) or large (housing 80 to 120 monkeys) corrals and were socially housed indoors either in pairs or in small social groups at the time of MRI data acquisition. Housing rooms were temperature-controlled with lights on at 6:00 AM and off at 6:00 PM and monkeys were fed monkey chow (LabDiet High Protein Monkey Diet; Ralston Purina) twice daily, supplemented with fresh fruit and vegetables twice weekly, and had ad libitum access to water. Monkeys were fasted beginning at 4:00 PM the day prior to MRI data acquisition. A subset of the subjects (N_male_ = 3; N_female_ = 13) were previously part of another study (103) and had been infected with Zika virus or exposed to monkeys who were infected with Zika virus, but had no active infection for at least 1 y prior to MRI data acquisition. These subjects had no clinical signs of infection during the acute infection phase (103). Zika virus infection is not known to cause any neurological abnormalities in healthy adults and no abnormalities were observed on MRI, so these subjects were considered neurologically normal for the purposes of the current experiments.

### Imaging Procedures.

Structural and functional MR images were acquired using a 3T Siemens Skyra scanner with a custom-built eight-channel head coil (Rapid MR International) optimized for monkey brain imaging. Monkeys were sedated with an initial dose of ketamine (5 mg/kg) and then endotracheally intubated prior to placement in an MR-compatible stereotaxic apparatus. A consistent level of anesthesia was maintained with isoflurane (~1.5%). Some monkeys (N = 16) had an IV line placed for the delivery of fluids prior to stereotaxic placement as multiparametric quantitative and diffusion MRI data were also acquired (thus requiring longer sedation and fluids). For monkeys who received IV fluids during data acquisition, catheter placement in the right vs. left leg was counterbalanced to prevent interference with the affective touch paradigm. Rectal temperature, respiration, end-tidal CO_2_, and SpO_2_ were monitored throughout imaging procedures. Body temperature was maintained with chemical heating pads. Isoflurane levels were adjusted according to physiological monitoring to ensure that monkeys did not wake up during the stimulation of the affective touch paradigm but were as light as possible in the stereotaxic frame to preserve functional networks. Functional scans were acquired last to ensure that the only active anesthetic agent was the isoflurane (rather than a combination of isoflurane and ketamine from the initial sedation). Isoflurane levels ranges from 1.3 to 1.8% during the affective touch paradigm (mean = 1.6%), heart rate ranged from 103 to 162 beats/min (mean = 126 bpm), and respiration rate ranged from 10 to 39 breaths/min (mean = 19.7 breaths/min).

T1-weighted structural volumes were acquired first with a Magnetization-Prepared Rapid Gradient-Echo (MP-RAGE) sequence (repetition time/echo time (TR/TE) = 2,500/3.65, voxel size = 0.3 × 0.6 × 0.6 mm^3^) followed by T2-weighted volumes (TR/TE = 3,000/308, voxel size = 0.4 × 0.8 × 0.8 mm^3^). Structural scans were then followed by multiparametric mapping and diffusion sequences for N = 16 monkeys prior to functional scans or were immediately followed by functional scans for the remaining N = 17. Four 10-min functional scans were acquired (T2*-weighted echo-planar sequence, TR/TE = 2,300/24, voxel size = 0.7 × 0.7 × 1.4 mm^3^) in two pairs each with reversed phase encode blips for distortion correction. During the first pair of scans, resting state data were acquired (used only for distortion correction in the present analyses). During the second pair of scans, monkeys were subjected to the affective touch paradigm.

### Affective Touch Paradigm.

The affective touch paradigm was designed to assess the neural networks responsive to interoceptive C-fiber mediated touch, contrasting these with tactile responses to discriminative touch. The paradigm was a block design, including 40 blocks per run, each lasting 15 s (for a total of 10 min). Each block belonged to one of three conditions: slow touch (3 cm/s), fast touch (15 cm/s), or rest (no touch). For the slow touch condition, 3 cm/s was selected according to prior work in humans which demonstrated that this speed optimally activates C-tactile afferents and produces the highest ratings of pleasantness (8). This speed has also been used frequently in other human studies of affective touch (e.g., refs. 26, 32, and 99), although we note that at least one early study in macaques found that C-fibers were optimally activated by touch in the range of 0.5 to 2 mm/s (5). For the fast touch condition, 15 cm/s was chosen because it is outside of the range of touch velocities that maximally activate C-fibers (8) and could be reliably delivered by a human experimenter in the scanner over the 15-s trials without introducing differences in stimulation pressure. Trial order was optimized to minimize variance in the design matrix using the fMRI Simulator tool (https://github.com/neurolabusc/fMRI-Simulator/tree/main) following the recommendations of ref. 104. During one run, stimulation was applied to the outer left thigh and during the other run stimulation was applied to the outer right thigh. The order of left vs. right leg stimulation and phase encoding direction during each scan was counterbalanced across subjects. A trained experimenter applied stimulation to the monkey’s leg. The experimenter wore a nitrile glove and provided stimulation with the open palmar surface of their hand. Stimulation speed was maintained according to visual cues (i.e., speed condition and time left in block) presented on a monitor at the edge of the scanner bore controlled by a custom Python script. A 15 cm piece of tape was placed adjacent to (but not touching) the monkey’s thigh as a reference for tracking stimulation velocity. The entire 15 cm region of thigh was stroked at a continuous velocity each 1 s (15 cm/s) during the fast condition and the region was stroked only 3 times (5 s per stroke, 3 cm/s) during the slow condition. Stimulation was provided in the direction of hair growth.

### fMRI Analysis.

#### Preprocessing.

Functional imaging data were processed with custom AFNI pipelines (105, 106). Raw images were first converted to the NIFTI file data format. T1- and T2-weighted images were processed using the CIVET-Macaque pipeline (107), which includes correction for contrast nonuniformities using N3 bias field correction and the generation of a brain mask. Normalized and skull-stripped T1-weighted images were then nonlinearly aligned to the NMT atlas (v2) using AFNI’s @animal_warper command (106, 108). Functional echoplanar imaging (EPI) data were preprocessed using a custom version of the AFNI NHP preprocessing pipeline (106, 109). Left and right leg stimulation data were processed separately using the same parameters. Images were slice time corrected, motion corrected, and aligned with the T1-weighted image and warped to the standard space. Distortion correction was accomplished using EPI data from the resting state scan with reverse phase encoding (i.e., reverse blip). Following alignment to standard space, EPIs were blurred using a 2-mm FWHM filter and rescaled to reflect percentage signal change from baseline. The hemodynamic response function was convolved with the three regressors of interest (fast, slow, rest) and six motion regressors of no interest using AFNI’s 3dDeconvolve. Four general linear tests were conducted, contrasting: slow vs. fast, slow and fast vs. rest, slow vs. rest, and fast vs. rest. The rest condition was treated as a baseline as no tactile stimulation was applied.

#### Group-level analysis.

Mixed effects meta-analysis was performed to determine whole-brain group-level differences in responses to slow vs. fast touch using AFNI’s 3dMEMA, which models both within- and between-subject variability. This analysis was performed on statistical maps obtained using AFNI’s 3dREMLfit, which conducts generalized least squares regression. The Hartung–Knapp adjustment was applied to the output *t*-statistic given the sample size. Age was included as a covariate in the mixed effects model. Left and right leg stimulation were modeled separately. For the purposes of visualization, maps of *t*-statistics are shown in *SI Appendix*, Fig. S4. All data are shown, with voxels meeting a threshold of *t* > 1.99 (corresponding to an uncorrected *P*-value of <0.05) and belonging to clusters of greater than 40 voxels highlighted using a black outline. Other voxels are shown with increasing transparency according to the decreasing value of the *t*-statistic. Primary analyses were conducted on ROI in the IAN and somatosensory cortices, described below.

#### Region of interest-based analyses.

Custom ROIs were drawn on the NMT reference brain [as we have done previously (110)] and resampled to the resolution of the fMRI data for ROI-based analyses of insula, ACC, and amygdala. ROIs were drawn for insula, ACC, and amygdala based on the established neuroanatomical literature. These regions were chosen due to a priori hypotheses about their involvement in processing affective touch. Separate ROIs were used for left and right hemisphere structures. The beta values for all voxels within each ROI were averaged, resulting in 1 beta value per hemisphere (left, right) per region per subject in each condition (fast, slow, rest). For ROI-based analyses of secondary somatosensory cortex, primary somatosensory cortex, and premotor cortex, we used the ARM atlas ROIs for these structures (106, 111). Linear mixed-effects models were used to analyze ROI data. These models were implemented in R version 4.3.1 (112) using the lmer function from the package lme4 (113). Mixed effects models included touch speed (fast vs. slow), hemisphere (left vs. right), age (continuous), and stimulation leg (left vs. right) as fixed effects and hemisphere nested within stimulation leg nested within subject as random effects. The model also included the interaction between touch speed and each of hemisphere, age, and leg (thus permitting the detection of lateralized effects in either the brain or body on responses to affective vs. discriminative touch and potential changes across the lifespan). Post hoc tests to assess interactions were conducted on the EMMs using the emmeans package (114). An α = 0.05 level of significance was used for all ROI-based analyses.

Post hoc analyses to determine functional specificity within subregions of insula were conducted using sliding ROIs over the extent of the structure. The whole insula ROI was truncated into four voxel-wide increments (6 mm, on the A–P axis), each advancing by one voxel (1.5 mm in template space). Average beta values for the slow and fast condition in each sliding ROI were then compared using a paired *t* test implemented with the *t* test function from the rstatix package (115). Loess regression was used to fit a smooth curve to the *t* statistics (as a function of location in the insula) and then subjected to a change point analysis using the cpt.mean function from the package changepoint (116). This function allows for the detection of a point (or points) at which the statistical properties of an ordered sequence of data change, highlighting potential heterogeneity in neural responses to affective vs. discriminative touch over the extent of the insula.

#### PPI analysis.

PPI analysis offers the opportunity to circumvent confounding factors [i.e., unconstrained mental activity and changes in signal-to-noise ratio (117)] and assess changes in functional connectivity related to task conditions (118, 119). A generalized form of the context-dependent PPI method [gPPI (57)] analysis was performed to assess task-based changes in functional connectivity with the right posterior insula. At the individual subject level, we generated interaction regressors by first extracting the average time series of the posterior insula ROI using AFNI’s 3dmaskave, then removing the trend from the time series using AFNI’s 3dDetrend, up-sampling the time series to 0.1 s TRs, running deconvolution on the seed time series using AFNI’s 3dTfitter (with a BLOCK basis function), and multiplying the deconvolved time series with separate up-sampled stimulus timing files for each condition (containing 1 for each TR during a given condition and 0 for the TRs during the other 2 conditions). These interaction regressors were then added into the regression analysis using AFNI’s 3dDeconvolve, with the primarily contrast of interest represented by the difference between the interaction regressors for the slow and fast conditions. Group analysis was then carried out using AFNI’s 3dttest.

## Supplementary Material

Appendix 01 (PDF)

## Data Availability

Data from all ROI-based analyses are available on the Open Science Framework at https://osf.io/4thgf/ (120). Raw imaging data will be publicly available via the PRIME-DE website (https://fcon_1000.projects.nitrc.org/indi/indiPRIME.html) after all data analyses are complete for this dataset and are available upon request.

## Footnotes

^*^We note that we did not evaluate the pleasantness of slow touch but we are calling slow touch affective because it activated the same neural network observed in humans during slow touch which has been rated as pleasant and previous work provides some evidence that slow touch is pleasant for monkeys (e.g., refs. 121 and 122). Future work should include dependent variables that track with the valence of experience in monkeys [e.g., high frequency heart rate variability (97, 98)].
