# Supplementary material for: Evolutionarily conserved neural responses to affective touch in monkeys transcend consciousness and change with age
Source: Proc Natl Acad Sci U S A. 2024 Apr 22;121(18):e2322157121. doi: 10.1073/pnas.2322157121 (PMC11067024; doi:10.1073/pnas.2322157121)
Supplement: Supplementary file 1 — Appendix 01 (PDF) [file pnas.2322157121.sapp.pdf]

Supporting Information for

Evolutionarily conserved neural responses to affective touch in monkeys transcend consciousness and change with age

Joey A. Charbonneau[1,2]*, Anthony C. Santistevan[2,3], Erika P. Raven[4], Jeffrey L. Bennett[2,3,5,6], Brian E. Russ[7,8,9], Eliza Bliss-Moreau[2,3]*

[a] Neuroscience Graduate Program, University of California Davis, Davis, CA, USA
[b] California National Primate Research Center, University of California Davis, Davis, CA, USA
[c] Department of Psychology, University of California Davis, Davis, CA, USA
[d] Center for Biomedical Imaging, Department of Radiology, New York University Grossman School of Medicine, New York, NY, USA
[e] Department of Psychiatry and Behavioral Sciences, University of California, Davis School of Medicine, Sacramento, CA, USA
[f] The MIND Institute, University of California Davis, Sacramento, CA, USA
[g] Center for Biomedical Imaging and Neuromodulation, Nathan Kline Institute, Orangeburg, NY, USA
[h] Nash Family Department of Neuroscience and Friedman Brain Institute, Icahn School of Medicine at Mount Sinai, New York, NY, USA
[I] Department of Psychiatry, New York University Langone, New York, NY, USA

* Correspondence:
Joey A. Charbonneau or Eliza Bliss-Moreau
jcharbonneau@ucdavis.edu or eblissmoreau@ucdavis.edu

This PDF file includes:

Figures S1 to S5

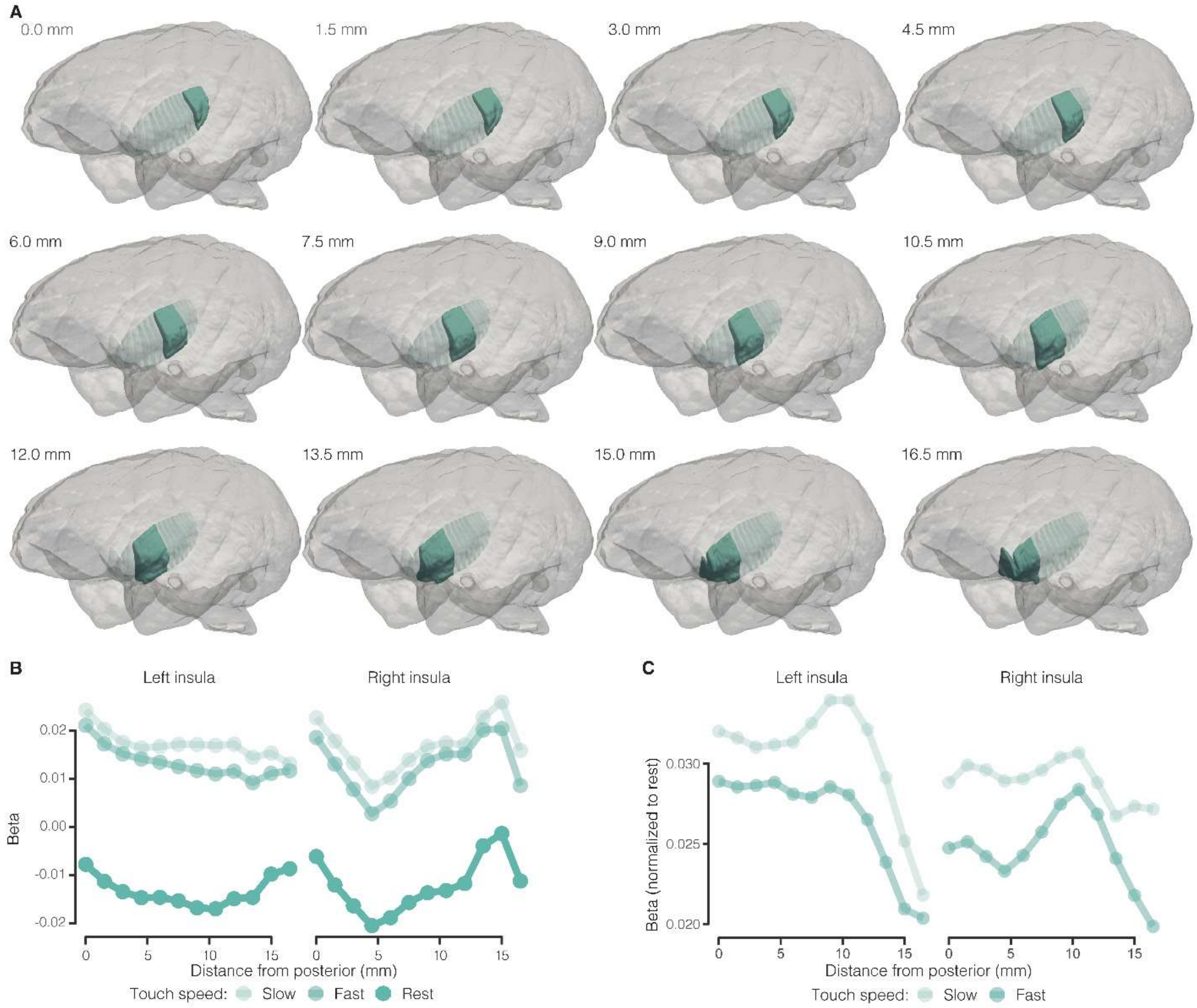

Figure S1.
Sliding regions of interest in the insula. **(A)** 3D renderings of sliding insula regions of interest. Only left insula ROIs are shown for visualization purposes; right insula ROIs were symmetric. The AP coordinates shown in the top right of each panel indicate the distance from the posterior extent of the whole insula ROI to the posterior extent of a given sliding ROI (and matching the x axis in **(B)** and **(C)**). Each ROI is 6mm in length on the AP axis (voxels are 1.5mm^3 in template space). **(B)**, Group-averaged beta values for each of the three conditions in each sliding insula ROI. Points with the greatest separation between slow (most transparent) and fast (middle transparency) correspond to the greatest *t*-statistic values in Main Text Fig. 1f. **(C)** Group-averaged beta values as in **(B)**, but shown as the difference between Slow – Rest and Fast – Rest.

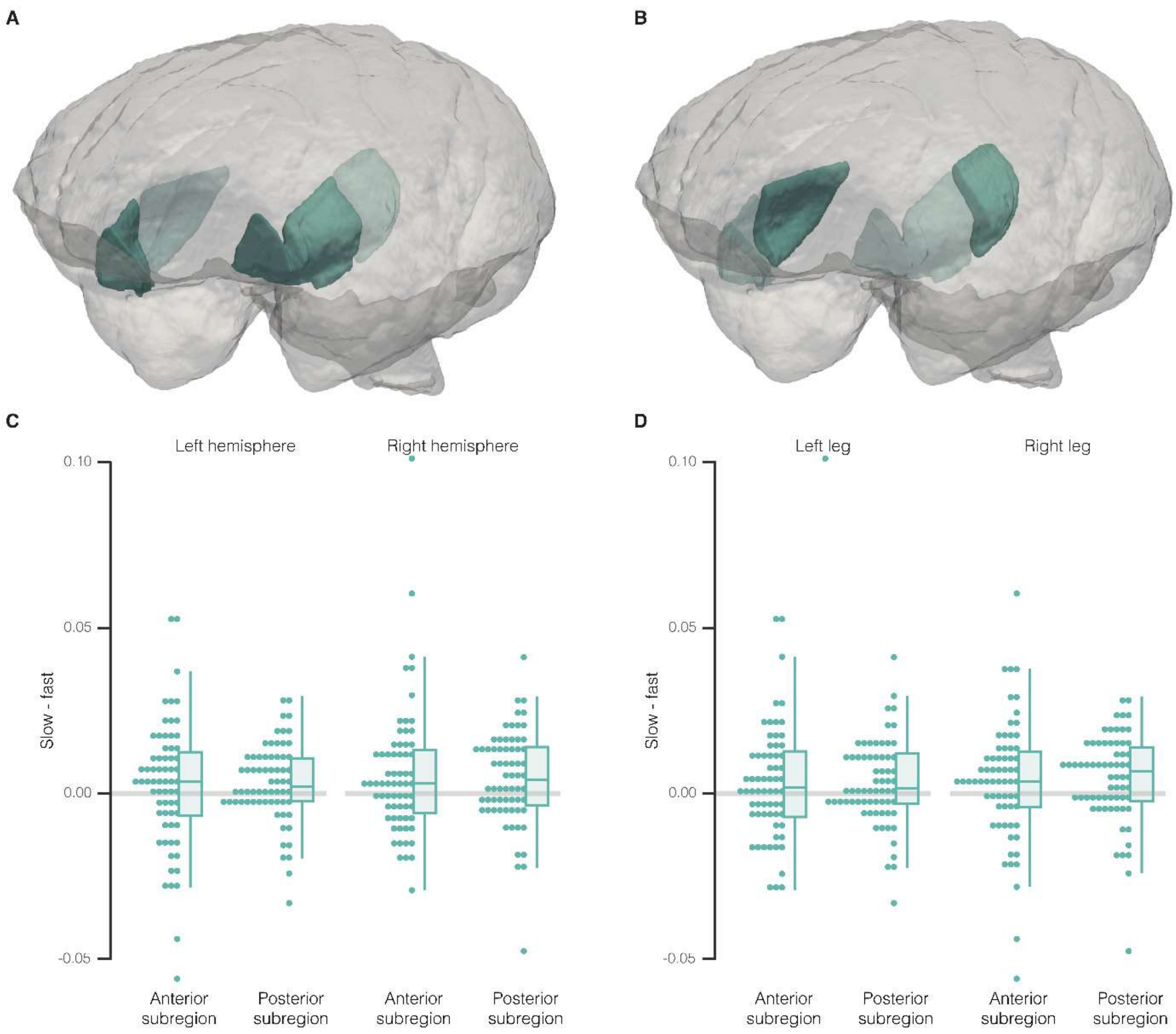

Figure S2.
Anterior vs. posterior insula subregion activation to slow vs. fast touch. **(A-B)** 3D visualization of the anterior (**A**) and posterior (**B**) insula ROIs. **(C-D)** Comparison of slow – fast condition activation in the left vs. right hemisphere (**C**) and following left vs. right leg stimulation (**D**) for the anterior and posterior insula subregions. At the group level, activation during slow touch was greater than activation during fast touch regardless of hemisphere, leg, or insula subregion.

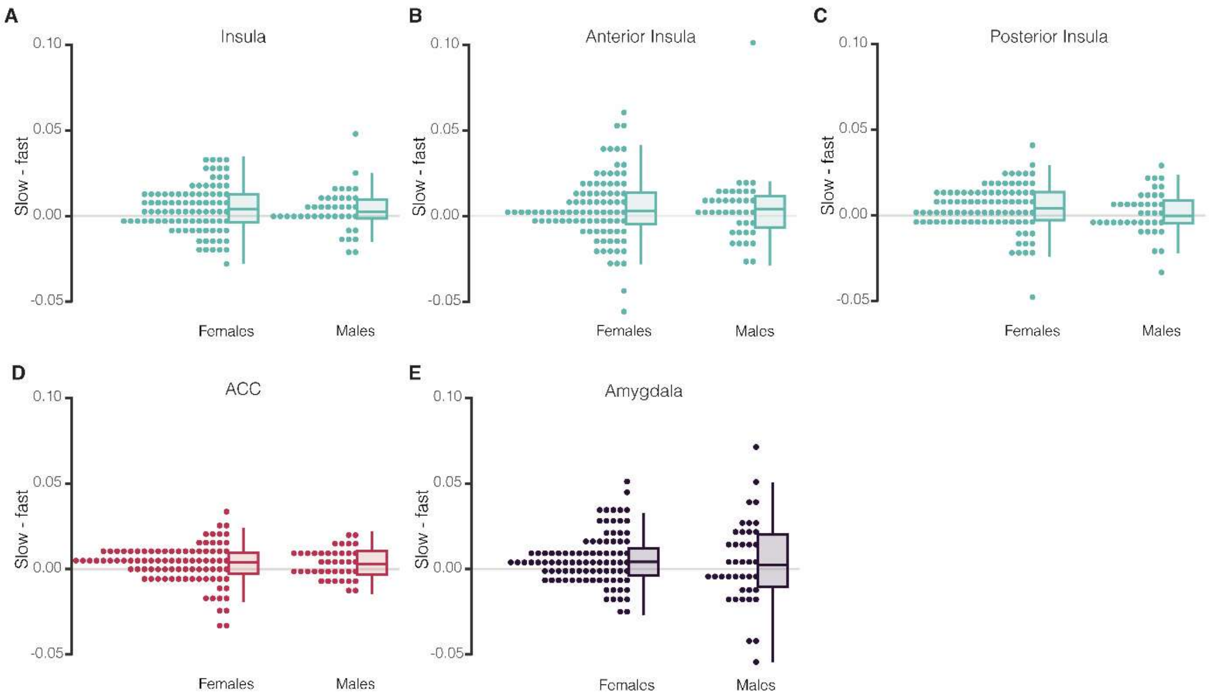

Figure S3.
Evaluation of sex differences in interoceptive-allostatic activation to Slow vs. Fast touch. **(A-E)** Separate visualizations of the distribution of slow – fast beta values for females (left) and males (right) in the insula (**A**), anterior insula (**B**), posterior insula (**C**), ACC (**D**), and amygdala (**E**). There were no sex differences in activation of any structure by slow vs. fast touch.

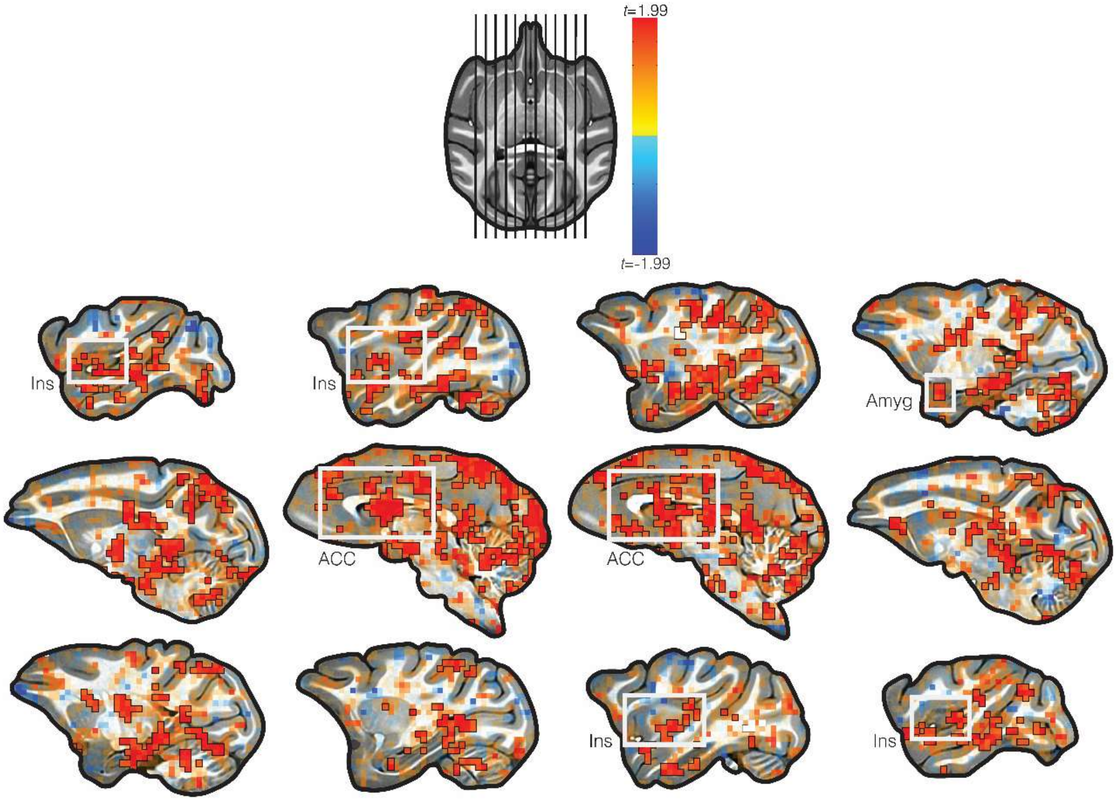

Figure S4.
Whole brain activation in response to slow vs. fast touch shown on a series of sagittal sections. Warm colored voxels (red) indicate greater responses during slow touch, cool colored voxels (blue) indicate greater responses during fast touch. Statistical maps are shown with a graded transparency such that increasing *t*-values are shown with greater opacity. Voxels with $t>1.99$ ($p<0.05$, uncorrected) belonging to clusters of a size greater than 40 contiguous voxels are outlined in black and shown with the greatest opacity. All other colors shown fall below this threshold. White outline boxes highlight significant voxels within interoceptive-allostatic network hub regions of interest (Ins: insula; ACC: anterior cingulate cortex; Amyg: amygdala).

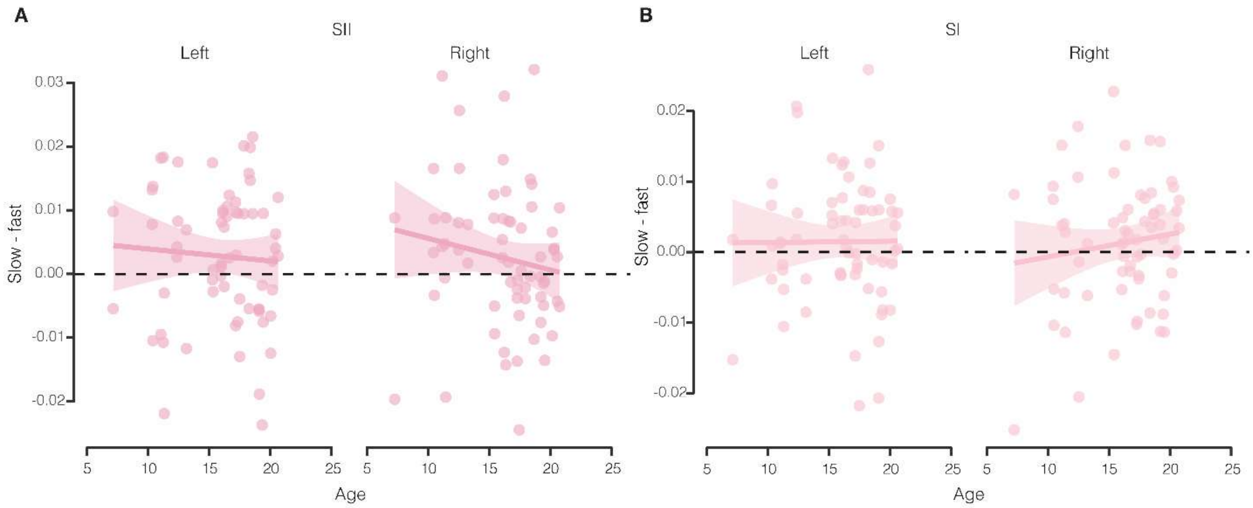

Figure S5.
Evaluation of age-related changes to activation in somatosensory regions. **(A-B)** Difference in beta values during slow vs. fast touch conditions as a function of age for secondary somatosensory cortex (**A**) and primary somatosensory cortex (**B**). There was no interaction between touch speed and age for either somatosensory region. Left vs. right hemisphere is shown separately for each region. Individual data points show the difference in activation across the ROI for slow – fast conditions.
